# Preventing and Managing Pre- and Postoperative Micronutrient Deficiencies: A Vital Component of Long-Term Success in Bariatric Surgery

**DOI:** 10.3390/nu17050741

**Published:** 2025-02-20

**Authors:** Claudia Reytor-González, Evelyn Frias-Toral, Cristina Nuñez-Vásquez, Juan Marcos Parise-Vasco, Raynier Zambrano-Villacres, Daniel Simancas-Racines, Luigi Schiavo

**Affiliations:** 1 Universidad UTE, Facultad de Ciencias de la Salud Eugenio Espejo, Centro de Investigación en Salud Pública y Epidemiología Clínica (CISPEC), Quito 170527, Ecuador; claudia.reytor@ute.edu.ec (C.R.-G.); cristina.nunez.v@gmail.com (C.N.-V.); juan.parise@ute.edu.ec (J.M.P.-V.); 2 Escuela de Medicina, Universidad Espíritu Santo, Samborondón 0901952, Ecuador; evelynft@gmail.com; 3 Division of Research, Texas State University, 601 University Dr, San Marcos, TX 78666, USA; 4 Escuela de Nutrición y Dietética, Universidad Espíritu Santo, Samborondón 0901952, Ecuador; rzambranovillacres@uees.edu.ec; 5 Department of Medicine, Surgery and Dentistry “Scuola Medica Salernitana”, University of Salerno, 84081 Baronissi, Italy

**Keywords:** obesity, bariatric surgery, micronutrient deficiencies, micronutrient supplementation, healthcare

## Abstract

Bariatric surgery (BS) is an effective treatment for severe obesity and its related comorbidities, such as type 2 diabetes and hypertension. However, the anatomical and physiological changes associated with these procedures significantly increase the risk of preoperative and postoperative micronutrient deficiencies, which can lead to severe complications such as anemia, osteoporosis, and neurological disorders. This narrative review examines the prevalence and clinical implications of micronutrient deficiencies in BS patients, as well as evidence-based strategies for their prevention and management. The most common deficiencies include iron, vitamin B12, folate, calcium, vitamin D, and fat-soluble vitamins (A, E, and K). Procedures with a hypoabsorptive component, such as Roux-en-Y gastric bypass (RYGB) and biliopancreatic diversion with duodenal switch (BPD/DS), pose higher risks of deficiencies compared to restrictive procedures like sleeve gastrectomy (SG). Effective strategies involve the preoperative correction of deficiencies, continuous monitoring, and tailored supplementation. However, long-term adherence to supplementation tends to decrease over time, influenced by behavioral and socioeconomic factors. Hence, preventing and managing micronutrient deficiencies are crucial for the long-term success of BS. While current guidelines provide valuable recommendations, many are based on low-certainty evidence, underscoring the need for more robust studies. A multidisciplinary approach, combined with innovative strategies, such as telemedicine, can enhance adherence and achieve sustainable clinical outcomes.

## 1. Introduction

Bariatric surgery (BS) has become a cornerstone in the management of obesity, particularly for patients who have not achieved significant or sustainable weight loss despite comprehensive non-surgical approaches, including dietary, behavioral, and pharmacological interventions. These procedures, such as Adjustable Gastric Banding (AGB) [1], Roux-en-Y gastric bypass (RYGB) [2], sleeve gastrectomy (SG), and biliopancreatic diversion with or without duodenal switch (BPD/DS) [3], not only facilitate substantial weight loss but also improve or resolve obesity-related comorbidities like type 2 diabetes, hypertension, and dyslipidemia [4,5]. Furthermore, BS is associated with improved survival rates and enhanced quality of life, solidifying its role as a transformative treatment option for severe obesity [6].

The American Society of Metabolic and Bariatric Surgery and the International Federation for the Surgery of Obesity and Metabolic Disorders recommend BS for patients with a body mass index (BMI) ≥ 35 kg/m^2^, regardless of the presence of comorbidities, and for those with a BMI of 30–34.9 kg/m^2^ when metabolic diseases are present or non-surgical treatments have failed. Following a multidisciplinary evaluation, it is also indicated for children and adolescents with a BMI > 120% of the 95th percentile and severe comorbidities, or >140% of the 95th percentile without comorbidities. Additionally, BS is effective in patients with extreme obesity (BMI > 60 kg/m^2^) and can serve as a bridge to optimize outcomes in procedures like organ transplants or other surgeries [7,8,9].

Beyond the potential complications [10,11], many candidates for BS enter the process with preexisting micronutrient deficiencies due to obesity-related factors [12,13]. Chronic inflammation, poor dietary quality, and metabolic imbalances common in individuals with obesity can result in deficiencies of iron, vitamin D, vitamin B12, folate, and zinc, even before surgery [13,14]. These deficiencies are often associated with specific comorbidities: for instance, vitamin D deficiency is linked to the development of metabolic bone diseases, such as osteoporosis or osteomalacia; vitamin B12 deficiency is associated with adverse lipid profiles and increased cardiovascular risk; and iron deficiency can lead to anemia, exacerbating fatigue and reducing exercise tolerance in individuals with obesity [13,14,15].

If left unaddressed, preoperative deficiencies can exacerbate postoperative risks, potentially leading to delayed recovery, poor wound healing, and complications such as anemia and osteoporosis [15].

The anatomical and physiological changes induced by BS can amplify existing micronutrient deficiencies or lead to new ones. Reduced dietary intake, bypassing of key absorptive sites, altered gastric pH, and disrupted enzyme and microbiota function are among the factors contributing to these risks [16]. Postoperative deficiencies commonly include iron, vitamin B12, folate, calcium, and vitamin D, with additional risks of deficiencies in zinc, thiamine (vitamin B1), and fat-soluble vitamins (A, E, and K) [17]. These deficiencies can result in a wide range of clinical complications, including anemia, neurological disorders, osteoporosis, and impaired immune function [18].

Proactive management of micronutrient status is critical throughout the bariatric care continuum. Preoperative screening and correction of deficiencies are vital to optimize surgical outcomes and minimize postoperative risks [19]. Following surgery, tailored supplementation and regular monitoring are essential to address evolving nutritional needs and ensure long-term health benefits [20].

This narrative review comprehensively examines both preoperative and postoperative micronutrient deficiencies in BS patients, their clinical implications, and evidence-based strategies for prevention and management. It aims to provide actionable insights for healthcare professionals to enhance patient care and achieve better outcomes.

## 2. Common Postoperative Micronutrient Deficiencies

Postoperative micronutrient deficiencies are a significant health concern for individuals undergoing BS, such as RYGB, SG, or BPD/DS. These procedures, designed to promote weight loss and alleviate obesity-related comorbidities, induce substantial anatomical and physiological alterations to the gastrointestinal tract [2]. RYGB involves bypassing the stomach, duodenum, and part of the jejunum, significantly reducing the absorptive surface for nutrients. SG removes approximately 80% of the stomach, leading to impaired gastric acid production, which affects iron and vitamin B12 absorption. BPD/DS causes the most severe malabsorption by bypassing a large portion of the small intestine, leading to major deficiencies in macronutrients and fat-soluble vitamins. At the histological level, the remaining intestinal mucosa undergoes adaptive changes, such as increased villus height and crypt depth, to compensate for reduced absorptive areas. Cellular alterations include modifications in brush border enzyme activity and nutrient transporter expression, which can further affect nutrient uptake. Molecularly, BS impacts gut hormones, with increased levels of glucagon-like peptide-1 and peptide YY, enhancing satiety and insulin secretion, while ghrelin levels, which regulate hunger, decrease, particularly after SG [2].

These changes impair the body’s ability to effectively absorb essential nutrients, potentially leading to deficiencies with serious health implications if not adequately managed [2].

**Figure 1:**
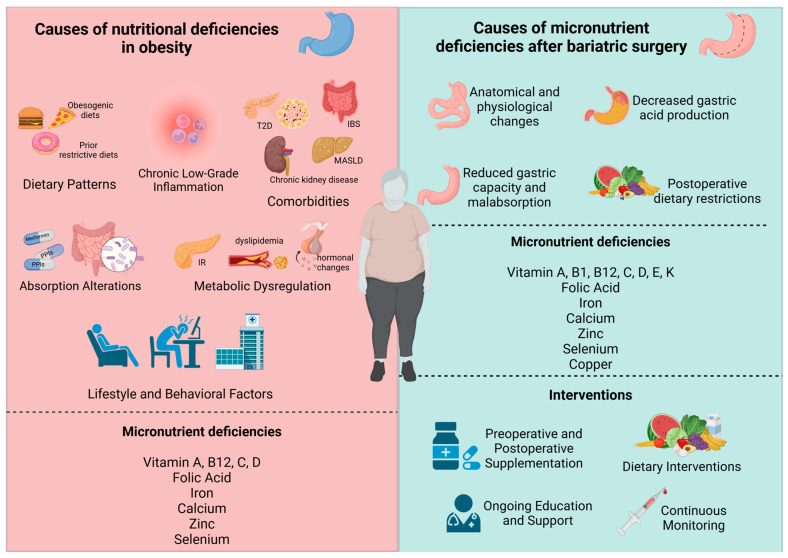
Determinants and Intervention Strategies for Nutritional Deficiencies in Obesity and Post-Bariatric Surgery. Causes of nutritional deficiencies in obesity and after bariatric surgery, highlighting the role of dietary patterns, comorbidities, metabolic dysregulation, and postoperative changes. Interventions include preoperative and postoperative supplementation, nutritional modifications, ongoing education, and continuous monitoring to address micronutrient deficiencies such as vitamins, iron, and calcium.

The mechanisms underlying nutritional deficiencies vary according to the type of BS performed [17,21]. Restrictive procedures like AGB primarily limit food intake, which may reduce the consumption of nutrient-dense foods, especially during the early postoperative period [2]. SG, which also reduces stomach size, impairs the digestion and absorption of key nutrients such as iron and vitamin B12 due to decreased gastric acid production [22]. Hypoabsorptive procedures, including BPD/DS, bypass large segments of the gastrointestinal tract, such as the duodenum and jejunum, leading to reduced nutrient absorption and significantly lower levels of macronutrients and fat-soluble vitamins [23]. RYGB combines both restrictive and hypoabsorptive mechanisms, with the degree of nutrient malabsorption often depending on the length of the Roux limb [21] (Figure 1).

A study by Côté et al. [21] evaluated micronutrient deficiencies across these three bariatric procedures, finding that BPD/DS patients faced the most significant challenges due to its extensive hypoabsorptive design. At baseline, 54.8% of BPD/DS patients exhibited vitamin D insufficiency, compared to 38.3% in SG and 39.9% in RYGB. Despite reductions in some deficiencies after 24 months of supplementation, BPD/DS patients consistently showed persistently lower levels of iron, calcium, and vitamin A compared to SG and RYGB. Additionally, BPD/DS patients required higher supplementation doses to maintain adequate vitamin A and D levels, underscoring the increased nutritional risks associated with this procedure. In contrast, SG was associated with fewer deficiencies, highlighting its comparatively lower impact on nutrient absorption [21].

The high prevalence of iron, vitamin B12, calcium, and vitamin D deficiencies among postoperative patients reinforces the necessity for lifelong nutritional monitoring and tailored management strategies [24,25]. These measures are crucial for minimizing complications, optimizing postoperative care, and improving long-term outcomes in BS patients.

### 2.1. Iron Deficiency

Iron plays an essential role in various physiological functions, particularly as a key component of hemoglobin in red blood cells and myoglobin in muscles, facilitating oxygen transport and storage [26]. Beyond its involvement in oxygen metabolism, iron is integral to enzymatic reactions, DNA synthesis, and energy production. Its presence in numerous iron-containing proteins highlights its necessity for maintaining systemic health [27]. Imbalances in iron levels are associated with conditions such as iron deficiency anemia, and iron overload diseases, such as hereditary hemochromatosis [28].

Iron deficiency is a prevalent condition among individuals with obesity, primarily due to a reduced capacity for iron absorption in the duodenum [29,30,31]. This pathophysiological mechanism has been demonstrated in various studies. For instance, a study by Mujica-Coopman et al. found a significantly lower iron absorption in women with obesity compared to those with overweight or normal weight (*p* < 0.05) [32]. Additionally, research has consistently reported that individuals with obesity face a higher risk of iron deficiency than those with normal weight. Schiavo et al. reported that 45% of subjects with obesity who were candidates for BS had iron deficiency [15,33,34].

BS, often performed to manage obesity, can exacerbate iron deficiency, affecting approximately 30% to 60% of patients postoperatively [2]. This heightened risk arises from a combination of physiological and dietary factors [35]. The bypass of the duodenum and proximal jejunum, the primary sites for iron absorption, significantly impairs the body’s ability to absorb this critical micronutrient. Moreover, reduced gastric acid production following surgery disrupts the conversion of dietary ferric iron (Fe^3^⁺) into its more bioavailable ferrous form (Fe^2^⁺) [36]. Postoperative dietary restrictions and intolerances further exacerbate this issue by limiting the consumption of iron-rich foods, such as red meat [37].

In addition, other contributing factors include Helicobacter pylori infection and bacterial overgrowth, which are more frequently observed in patients after BS. These conditions can impair iron absorption by interfering with gut integrity and function. Chronic gastrointestinal blood loss, also commonly reported in these patients, further compounds the risk of iron deficiency [2]. Together, these multifactorial contributors underscore the critical importance of proactive monitoring and comprehensive management strategies to maintain adequate iron levels in BS patients.

Supporting these findings, a study involving 32 women who underwent BS and subsequent abdominoplasty revealed significant declines in hemoglobin and iron levels post-surgery. Hemoglobin levels decreased from 12.98 g/dL to 10.8 g/dL within 48 h, with partial recovery to 11.53 g/dL by the first postoperative week. However, serum iron and transferrin saturation remained persistently low, and ferritin levels showed a marked decline by the eighth week. Notably, 45% of the patients developed iron deficiency, emphasizing the critical need for vigilant monitoring and proactive management of iron levels in bariatric patients, particularly those undergoing additional surgical interventions [38].

Additionally, various studies have shown that iron deficiency and the resulting anemia can persist long-term after BS, particularly among women and patients who undergo RYGB, who are at higher risk [21,39]. A study conducted in Brazil revealed that even after a decade, anemia persisted in up to 45% of patients [40], while a Portuguese study reported a prevalence of 24.4% following four years of follow-up [41]. These findings highlight the critical importance of rigorous monitoring and continuous management of iron levels in this population.

Common clinical presentations of iron deficiency anemia (IDA) in patients with obesity include low hemoglobin levels (below 13 g/dL for men and 12 g/dL for women), transferrin saturation (TSAT) below 20%, and ferritin concentrations below 30 ng/mL, typically without signs of inflammation [35,42]. Symptoms such as chronic fatigue are often significant clinical indicators. Additionally, some patients may present with IDA without microcytosis due to coexisting vitamin B12 or folate deficiencies [22,43].

The impact of anemia on postoperative outcomes in BS patients is significant and multifaceted. Preoperative anemia and iron deficiency are strongly associated with higher rates of postoperative complications, such as infections and an increased need for blood transfusions [44]. These complications not only elevate morbidity and mortality but also adversely impact the healing process and the patients’ overall quality of life. Additionally, the presence of anemia can lead to prolonged hospital stays and greater healthcare costs [2].

Interestingly, even subclinical iron deficiency (without overt anemia) has been shown to impair postoperative recovery, contributing to symptoms like fatigue and reduced functional capacity [44]. These findings emphasize the critical role of thorough preoperative screening and proactive management of iron levels to mitigate these risks and improve recovery outcomes for BS patients.

### 2.2. Vitamin B12 and Folate Deficiencies

BS induces anatomical and physiological changes that predispose patients to significant micronutrient deficiencies, particularly vitamin B12 and folate [45]. These deficiencies not only negatively impact health but can also complicate the postoperative recovery process. The underlying mechanisms are related to surgical alterations combined with dietary and metabolic factors [46].

Vitamin B12 deficiency is primarily associated with disruptions in gastric and upper intestinal physiology. Its absorption requires two key components: an acidic environment in the stomach to release vitamin B12 from dietary proteins and intrinsic factor (IF), a glycoprotein produced by gastric parietal cells essential for absorption in the terminal ileum [47]. Procedures such as SG, RYGB, and BPD/DS negatively affect both these processes. Studies have reported vitamin B12 deficiency in up to 50% of patients undergoing RYGB [48] and 20% of those undergoing SG [49].

Reduced gastric acid production and the partial or total removal of parietal cells limit the formation of the vitamin B12–IF complex, leading to poor absorption in the distal ileum. This is further exacerbated by decreased consumption of B12-rich foods, such as meat and dairy, which are often restricted in postoperative diets [50]. A study by Lombardo et al. investigated the prevalence and persistence of vitamin B12 deficiency in BS patients, even among those adhering to supplementation protocols. They found that 15.4% of participants exhibited deficiency, with a higher prevalence in women (16.6%) than in men (10%) [47]. In contrast, the prevalence in the general population under 60 is approximately 6%, underscoring the insufficiency of standard postoperative protocols in preventing long-term deficiencies. These findings highlight the critical need for tailored nutritional strategies and lifelong monitoring in this patient population.

Vitamin B12 deficiency symptoms can develop insidiously since the liver stores significant amounts of this vitamin, sufficient for approximately two years [51]. Once depleted, symptoms may include fatigue, pallor, glossitis, peripheral neuropathy (numbness and tingling in extremities), and neuropsychiatric disturbances such as memory loss and depression [52]. Severe cases can result in megaloblastic anemia and irreversible neurological damage, such as ataxia and subacute dementia [47,50,53]. Early recognition of these symptoms and proactive management are essential to preventing severe complications and ensuring optimal patient outcomes.

Folate absorption occurs primarily in the proximal small intestine, particularly the jejunum, a region often bypassed in procedures like RYGB and BPD/DS [54]. Unlike vitamin B12, folate absorption does not require IF; however, surgical modifications such as hypochlorhydria, altered intestinal pH, and reduced absorptive surface area significantly impair its uptake. Additionally, postoperative diets often lack folate-rich foods like leafy greens and legumes [55,56].

Short-term dietary inadequacy quickly affects serum folate levels, with measurable declines occurring within three weeks [57]. Serum folate is an unreliable marker of overall folate status, as it reflects recent intake rather than tissue reserves [56]. Red blood cell folate levels decrease over several months and provide a more accurate indicator of long-term folate stores. Postoperative deficiencies range from 9% to 39%, depending on procedure type and dietary habits [58].

Folate deficiency primarily affects tissues with high cellular turnover, such as the hematopoietic system. Common symptoms include fatigue, pallor, glossitis, and megaloblastic anemia, which mimics vitamin B12 deficiency [46]. Additionally, low folate levels are associated with elevated plasma homocysteine, a risk factor for cardiovascular disease. For women of reproductive age, folate deficiency poses significant reproductive risks, including an increased likelihood of neural tube defects such as spina bifida and anencephaly in offspring [46,59].

A critical consideration in managing these deficiencies is their interaction. Both nutrients are essential for DNA synthesis and red blood cell production, and deficiencies in either can result in similar hematological outcomes, such as megaloblastic anemia. However, folate supplementation alone can mask the hematological symptoms of vitamin B12 deficiency, allowing neurological complications to progress undetected [60]. This highlights the importance of comprehensive evaluations of hematinic levels—including vitamin B12, folate, and iron—before initiating supplementation. Tailored management strategies are essential to optimize postoperative outcomes and prevent the long-term complications of folate deficiency.

### 2.3. Calcium and Vitamin D Deficiencies

Among the micronutrient deficiencies arising after BS, vitamin D and calcium deficiencies are particularly critical due to their essential roles in bone health and long-term implications [61,62,63]. These nutrients are closely interconnected, as vitamin D facilitates calcium absorption in the gut and supports bone mineralization, while calcium is vital for maintaining skeletal integrity. Disruptions in their absorption mechanisms following bariatric procedures place patients at significant risk for complications such as secondary hyperparathyroidism (sPTH), metabolic bone disease, and fractures [62].

Vitamin D is primarily absorbed in the jejunum and ileum through passive diffusion, a process dependent on bile salts rather than dietary fat. BS disrupts gastrointestinal anatomy, reducing the efficiency of this process. In RYGB, bile and pancreatic secretions mix with the food bolus only in the distal jejunum, limiting nutrient exposure to optimal absorption conditions. Similarly, SG reduces nutrient exposure to digestive enzymes due to decreased stomach capacity and altered flow dynamics [21].

Calcium absorption, which predominantly occurs in the duodenum and proximal jejunum, is severely impacted by RYGB, as these regions are bypassed. Reduced gastric acid production post-surgery and the use of proton pump inhibitors further hinder calcium solubilization, a critical step for absorption. Even in SG, where the intestinal anatomy remains intact, calcium absorption can be impaired due to reduced gastric acid secretion and caloric intake. Longitudinal studies have demonstrated significant reductions in calcium bioavailability in patients following these procedures [62].

Deficiencies in vitamin D and calcium often lead to sPTH, characterized by elevated parathyroid hormone (PTH) levels that increase bone resorption to maintain serum calcium levels. This compensatory mechanism accelerates bone turnover and decreases bone mineral density (BMD). Within the first-year post-surgery, studies have reported substantial declines in BMD, particularly in the hip (8–11%) following RYGB, with less pronounced reductions in spinal BMD [22].

Over time, these deficiencies contribute to metabolic bone disease, encompassing conditions like osteomalacia, osteoporosis, and heightened fracture risk. Krzizek et al. (2021) reported that up to 70.8% of BS patients experience vitamin D deficiency within a year of surgery, even with supplementation [17]. Similarly, Lombardo et al. emphasized that calcium malabsorption persists long-term, necessitating ongoing monitoring and intervention [47].

The interplay between vitamin D and calcium deficiencies exacerbates their effects on bone health. Vitamin D deficiency impairs calcium absorption in the gut, increasing reliance on bone resorption to maintain serum calcium levels. This cycle weakens the skeleton and elevates fracture risk, particularly in weight-bearing bones like the hip and vertebrae. Elevated PTH levels associated with sPTH further accelerate bone loss, disproportionately affecting the trabecular bone, which is more metabolically active and susceptible to resorption [62].

Effective management of these deficiencies requires comprehensive monitoring of serum 25-hydroxyvitamin D, calcium, and PTH levels. Regular follow-up and dose adjustments are critical to ensuring that patients meet their nutritional needs and prevent the progression of bone disease [47].

### 2.4. Other Micronutrient Deficiencies

Micronutrient deficiencies are a well-documented consequence of BS due to the extensive anatomical and physiological changes that impact nutrient absorption. Beyond commonly discussed nutrients like iron, vitamin B12, and calcium, deficiencies in zinc, thiamine, and fat-soluble vitamins (A, E, and K) can also occur, with significant clinical implications [22]. These deficiencies arise from a combination of reduced dietary intake, altered absorption mechanisms, and disrupted metabolic pathways.

- Zinc deficiency

Zinc plays a critical role in numerous biological processes, including protein synthesis, wound healing, and immune function. Its absorption primarily occurs in the proximal small intestine, a region often bypassed in hypoabsorptive surgeries such as RYGB and BPD/DS [64,65]. Reduced gastric acid production further diminishes zinc solubilization and absorption. These alterations, compounded by dietary restrictions post-surgery, lead to a high prevalence of zinc deficiency in bariatric patients. Clinically, zinc deficiency presents with nonspecific symptoms such as alopecia, delayed wound healing, and immune dysfunction. Severe deficiency can result in taste changes, dermatitis, and increased susceptibility to infections. Stein et al. emphasized that postoperative zinc deficiency can prolong recovery time and impair immune responses, particularly in patients with comorbidities [66].

- Thiamine (Vitamin B1) deficiency

Thiamine deficiency is a common early complication following BS, particularly RYGB and SG [67]. This water-soluble vitamin has limited body stores, which are rapidly depleted during periods of reduced intake or increased demand. Postoperative vomiting, a frequent complication after BS, further exacerbates thiamine depletion. Additionally, altered gastric pH and bypass of the duodenum reduce its absorption. Clinically, thiamine deficiency can manifest as Wernicke’s encephalopathy, characterized by confusion, ataxia, and ophthalmoplegia [68,69]. If untreated, this condition can lead to Korsakoff syndrome and irreversible neurological damage. Peripheral neuropathy and cardiovascular dysfunction (beriberi) are also associated with prolonged deficiency, for that reason, early detection is important for preventing these severe outcomes [70].

Fat-soluble vitamins (A, E, K)

- Vitamin A

Vitamin A is a fat-soluble nutrient essential for various physiological processes, including vision, immune function, maintenance of epithelial integrity, and embryonic development [2]. Additionally, it plays a critical role in regulating adipose tissue function. A significant proportion of the body’s retinoid content, approximately 15–20%, is stored in adipose tissue as retinol and retinyl esters, interacting with retinoid receptors to mediate metabolic processes. Interestingly, individuals with higher body fat levels tend to store greater amounts of fat-soluble vitamins, including vitamin A, in adipose tissue. However, studies have consistently shown lower serum concentrations of retinol in individuals with overweight and obesity compared to those with normal weight [71]. This phenomenon is likely due to the sequestration of vitamin A in adipose tissue, limiting its availability for physiological functions. Moreover, a negative correlation between serum retinol levels and BMI has been observed in various studies. After BS, vitamin A deficiency is prevalent, particularly within the first-year post-surgery [20,22]. This deficiency is compounded by reduced dietary intake, malabsorption, and altered metabolism. Beyond its well-known roles, vitamin A regulates gene expression related to metabolism and adipose tissue activity, extending its influence on energy balance and metabolic health. These findings emphasize the need for a comprehensive evaluation of vitamin A status, as serum levels alone may not fully reflect its bioavailability or functional sufficiency [72].

- Vitamin E

Vitamin E is a crucial antioxidant that protects cellular membranes from oxidative damage by scavenging free radicals. It plays a significant role in immune function, neurological health, and cellular signaling [73]. In patients with obesity, chronic low-grade systemic inflammation and increased oxidative stress elevate the demand for antioxidants like vitamin E. However, serum levels of this vitamin are often reduced in these individuals, potentially due to sequestration in adipose tissue, which limits systemic availability [74,75]. After BS, particularly hypoabsorptive procedures like BPD/DS, vitamin E deficiency is more prevalent due to impaired fat absorption [76,77]. Clinical manifestations include peripheral neuropathy, ataxia, muscle weakness, and increased susceptibility to infections. Monitoring serum α-tocopherol levels, adjusted for lipid concentrations, is essential for accurate assessment. Although deficiencies are rare after procedures like SG or RYGB, patients undergoing BPD/DS require routine monitoring to mitigate risks of oxidative stress and long-term neurological damage [77].

- Vitamin K

Vitamin K is crucial for blood clotting and bone health, as it activates clotting factors and regulates calcium deposition in bones [78]. In individuals with obesity, higher adiposity may increase the storage of fat-soluble vitamins, including vitamin K, in adipose tissue. However, serum levels are often reduced due to limited bioavailability. Deficiency in vitamin K can result in impaired blood clotting, prolonged bleeding, and reduced bone mineral density, thereby increasing fracture risk [67,79]. Although few studies specifically document vitamin K deficiencies following metabolic weight-loss surgery, available data suggest that hypoabsorptive procedures like BPD/DS are associated with higher prevalence rates. Studies report vitamin K deficiencies in up to 69% of patients undergoing BPD/DS [76,80,81].

These deficiencies highlight the importance of proactive monitoring and tailored care for BS patients, particularly those undergoing hypoabsorptive procedures, to address the systemic and long-term effects of insufficient zinc, thiamine, and fat-soluble vitamin levels.

## 3. Supplementation Strategies

### 3.1. Preoperative Supplementation

A comprehensive nutritional evaluation before BS is essential for identifying and addressing micronutrient deficiencies and malnutrition, which are prevalent among candidates due to suboptimal dietary patterns, metabolic alterations, or limited nutrient intake [82,83]. Research indicates that up to two-thirds of patients scheduled for BS exhibit malnutrition, underscoring the importance of preoperative screening [84]. Deficiencies in key nutrients such as vitamin D, calcium, thiamine, vitamin B12, folate, iron, zinc, copper, and fat-soluble vitamins (A, E, K) must be corrected to minimize surgical risks and enhance postoperative recovery [83,85]. These evaluations are particularly important for patients undergoing hypoabsorptive procedures like RYGB or BPD/DS, which exacerbate the likelihood of nutrient malabsorption.

#### 3.1.1. Assessment Tools and Biomarkers

The accurate assessment of nutritional status integrates validated screening tools with laboratory tests. Commonly used methods include the Subjective Global Assessment, which evaluates dietary history, weight changes, and physical signs of malnutrition to categorize patients as well-nourished, moderately malnourished, or severely malnourished [86,87]. The Nutritional Risk Index predicts surgical complications by analyzing serum albumin levels and weight changes [88]. The Mini Nutritional Assessment, along with its short-form variant, assesses factors like BMI and psychological conditions, particularly in older adults [86,89]. Other tools include the Malnutrition Universal Screening Tool, based on BMI, weight loss, and reduced food intake to evaluate nutritional status [90,91], and the Nutritional Risk Screening 2002, which evaluates malnutrition risk in hospitalized patients [92,93]. Additionally, the Geriatric Nutritional Risk Index uses albumin levels and weight relative to ideal weight to identify elderly patients at risk of malnutrition-related complications [94].

Each of these tools contributes to a holistic assessment of nutritional status, ensuring that BS candidates are thoroughly evaluated and optimally prepared for the procedure.

Biochemical markers complement other assessment tools by providing objective insights into a patient’s nutritional status [95,96]. Albumin and prealbumin are critical markers for evaluating protein reserves. Prealbumin, with its shorter half-life, is particularly valuable for detecting acute changes, whereas albumin reflects more chronic nutritional states. Both are essential for understanding protein-energy malnutrition [96,97]. C-reactive protein (CRP) is another important marker, measuring inflammation that can confound the interpretation of other nutritional markers, such as albumin and prealbumin. Elevated CRP levels indicate inflammatory states that may obscure underlying nutritional deficits [98].

Hemoglobin, ferritin, and iron levels are key indicators for diagnosing anemia and assessing iron stores, which are especially important for bariatric patients, as anemia is a frequent preoperative concern [99,100,101]. Similarly, vitamin D, vitamin B12, and folate levels require regular evaluation, as deficiencies in these micronutrients are common in bariatric patients. Addressing these deficiencies is vital for maintaining bone health, neurological function, and hematological stability [96,101]. Zinc and copper levels are also critical to assess, particularly for patients undergoing hypoabsorptive surgeries, as deficiencies in these trace elements are common. Zinc is essential for immune function and wound healing, while copper plays a vital role in various enzymatic processes [64,65,102].

Together, these complementary methods enhance the ability to identify subtle deficits and physiological imbalances, enabling a more comprehensive evaluation that supports tailored nutritional interventions for BS candidates.

#### 3.1.2. Recommended Preoperative Supplementation

Preoperative micronutrient supplementation is essential for addressing widespread nutritional deficiencies in BS candidates. Many patients present with inadequate nutrient levels due to poor dietary quality and metabolic alterations associated with obesity. Correcting these deficiencies is crucial not only for minimizing perioperative risks but also for improving recovery and long-term outcomes. Initiating supplementation 8–10 weeks before surgery provides sufficient time to restore nutrient balance and prepare the body for the physiological demands of the procedure [46].

Vitamin D deficiency, affecting over two-thirds of patients, significantly compromises bone health and immune function [103]. Its role in the immune response includes modulating the activity of T cells, enhancing antimicrobial peptide production, and regulating inflammatory cytokines. Low vitamin D levels have been associated with increased susceptibility to infections and autoimmune disorders. High-dose supplementation of cholecalciferol (4000 to 6000 IU/day) is effective for repletion, followed by maintenance doses of 2000 IU/day postoperatively [62]. This intervention is often paired with calcium citrate (1200–1500 mg/day) to ensure adequate calcium levels and prevent complications such as secondary hyperparathyroidism [62].

Iron deficiency, frequently associated with anemia, requires supplementation with 100–200 mg of elemental iron daily, alongside vitamin C (250–500 mg/day) to enhance absorption [46]. For patients unable to tolerate oral iron, intravenous formulations are recommended. Similarly, vitamin B12 is commonly deficient due to absorption challenges and is replenished through intramuscular injections of 1000 µg every two weeks or high-dose oral alternatives. Folate supplementation (2 mg/day) is also critical, particularly for patients presenting with anemia [103].

Thiamine (vitamin B1) supplementation (100 mg/day), is essential for patients experiencing poor intake or vomiting to prevent severe complications such as Wernicke’s encephalopathy. Fat-soluble vitamins (A, E, and K) should be supplemented based on specific deficiencies, with close monitoring, especially in those undergoing hypoabsorptive surgeries [46].

Regular monitoring of biochemical markers and individualized supplementation strategies tailored to the unique needs of each patient ensure effective repletion of these critical nutrients (Table 1). This comprehensive approach lays the groundwork for safer surgeries, reduced postoperative complications, and improved long-term health outcomes, enabling patients to achieve sustainable benefits from bariatric procedures.

#### 3.1.3. Preoperative Dietary Strategies

In addition to micronutrient supplementation, dietary interventions also play a crucial role in preoperative optimization for patients with obesity undergoing BS. Among these interventions, very low-calorie ketogenic diets (VLCKDs), recently known as Very Low Energy Ketogenic Therapy (VLEKT) [104], have emerged as a pivotal strategy in managing preoperative nutritional and metabolic optimization for these patients [105]. These diets are characterized by a strict reduction in carbohydrate intake to less than 50 g/day, moderate protein levels, and a high proportion of fat, inducing a ketogenic state that promotes rapid fat loss while preserving lean body mass [106]. By shifting the body’s energy metabolism to rely primarily on ketones derived from fat, VLCKDs have demonstrated substantial benefits not only in weight reduction but also in addressing metabolic dysfunctions such as hepatic steatosis, insulin resistance, and dyslipidemia, which are common in BS candidates [85,107,108,109]. Furthermore, the nutritional design of these diets can incorporate targeted micronutrient supplementation, addressing the deficiencies prevalent in this population.

Evidence supports the use of VLCKDs during the preoperative period for BS patients, highlighting their significant metabolic and nutritional benefits. Schiavo et al. have conducted a series of studies that illustrate the impact of VLCKDs in the preoperative period. A study evaluating a ketogenic diet enriched with micronutrients (1200 calories/day; macronutrient distribution: 4% carbohydrates, 71% fats, 25% proteins) showed significant reductions in body weight (10.3% in men and 8.2% in women, *p* < 0.001) and a notable decrease in the volume of the left hepatic lobe (−19.8%, *p* < 0.001) [25]. These improvements are particularly advantageous in reducing surgical complexity and enhancing the safety of laparoscopic procedures by improving hepatic and visceral fat profiles.

Another study by Schiavo et al. compared VLCKDs with standard low-calorie diets (LCDs) in patients with intragastric balloons. VLCKDs were found to preserve lean mass more effectively (3.55% vs. 14.3%, *p* < 0.001) and caused a smaller reduction in resting metabolic rate (9.79% vs. 11.4%, *p* < 0.001). While fat loss was more pronounced in the VLCKD group (41.6% vs. 33.1%), the difference did not reach statistical significance (*p* = 0.0606), further highlighting the metabolic advantages of ketogenic approaches over traditional LCDs [110].

Barrea et al. further supported the broader role of VLEKTs in reducing hepatic and visceral fat, facilitating safer laparoscopic procedures, and enhancing postoperative metabolic recovery. Compared to standard VLCDs, VLCKDs achieved superior weight loss, hepatic volume reduction, and overall metabolic improvement, emphasizing their safety and efficacy as a preoperative intervention [111].

These benefits extend beyond weight management [112]. Schiavo et al. demonstrated that combining VLCKDs with continuous positive airway pressure significantly improved obstructive sleep apnea syndrome, a common comorbidity among BS patients [113]. Additionally, their investigation into supplementation strategies showed that enriching whey protein with amino acids and vitamin D substantially preserved lean mass and muscle strength during the postoperative period in SG patients [114]. Collectively, the dual benefits of VLCKDs and targeted micronutrient supplementation in addressing the complex nutritional needs of BS candidates. Integrating these dietary strategies into preoperative care enables clinicians to mitigate micronutrient deficiencies, optimize metabolic profiles, and improve surgical outcomes.

While VLEKT may offer short-term benefits in weight reduction and metabolic improvement, its highly restrictive nature poses challenges in terms of long-term adherence and rebound risk. More studies are needed to evaluate these aspects, allowing healthcare professionals to consider their implications in the comprehensive management of patients. A better understanding of adherence and the maintenance of long-term results will ensure that these diets are used effectively and safely within a personalized nutritional approach.

Tailored interventions such as VLCKDs, validated screening tools, and individualized supplementation plans further enhance patient readiness for surgery, reduce surgical risks, and support long-term recovery, ultimately establishing a strong foundation for improved postoperative health and quality of life while ensuring BS candidates are fully prepared for the metabolic and physiological demands of the procedure.

### 3.2. Immediate Postoperative Supplementation

Immediate postoperative supplementation is critical to mitigate the heightened risk of nutritional deficiencies following BS. Several authors, including O’Kane et al. [46], and Parrott et al. [67], emphasize that comprehensive multivitamin and mineral supplementation should be initiated immediately and customized to the particular type of BS. All BS patients should receive supplementation that includes thiamine, iron, zinc, copper, and selenium. The recommended minimum daily intake is 2 mg of copper and 15 mg of zinc [46]. For hypoabsorptive procedures such as BPD/DS, higher doses of zinc, up to 30 mg/day, are recommended to maintain the appropriate zinc-to-copper ratio.

High-dose iron supplementation, such as 200 mg of ferrous sulfate daily (or equivalent), is also advised, with menstruating women requiring double doses to meet their increased needs. Vitamin C supplementation is recommended to enhance iron absorption [37]. Both O’Kane et al. and Parrott et al. stress the importance of separating iron and calcium supplementation by at least two hours to avoid absorption interference [46,67].

Vitamin D supplementation recommendations are consistent among experts, with daily doses of 2000–4000 IU suggested to maintain serum levels above 75 nmol/L. These doses may need to be adjusted upward for patients undergoing BPD/DS [39,57,107]. Similarly, calcium supplementation guidelines recommend 1200–1500 mg/day for SG and RYGB patients, increasing to 2400 mg/day for BPD/DS. Calcium citrate is preferred due to its superior bioavailability [46,67].

Vitamin B12 supplementation is strongly advised for BS patients, with intramuscular injections of 1 mg every three months for SG, RYGB, and BPD/DS patients, and more frequent administration if neurological symptoms develop. Thiamine supplementation should start at 12 mg/day through multivitamins, increasing to 200–300 mg/day in critical cases such as Wernicke’s encephalopathy [46,67].

For fat-soluble vitamins, O’Kane et al. recommend 10,000 IU/day for vitamin A [46], while Homan et al. suggest higher starting doses of 50,000 IU/day for BPD/DS patients, adjusted based on clinical monitoring [45]. Regarding vitamin E, Slater et al. suggest 60 IU/day [115], while Aills et al. recommend 300 µg/day for vitamin K in BPD/DS patients [116]. Water-miscible formulations of fat-soluble vitamins are preferred to enhance absorption, particularly in hypoabsorptive procedures. Selenium supplementation is also universally recommended, with doses adjusted based on deficiency detection through regular monitoring [45,117].

This comparative analysis highlights a consensus among experts while underscoring the importance of individualized supplementation protocols tailored to the specific surgical procedure. Routine monitoring and dose adjustments are essential to prevent long-term complications and ensure optimal nutritional status for BS patients [45,46,67] (Table 2).

### 3.3. Long-Term Supplementation

Long-term micronutrient deficiencies are critical for patients following BS, necessitating continuous supplementation and rigorous monitoring to prevent complications. Ben-Porat et al. conducted a comprehensive four-year follow-up study on SG patients, revealing persistent challenges in maintaining adequate nutrient levels [52]. Despite initial postoperative improvements, vitamin D deficiency remained strikingly high, affecting 86.4% of patients after four years. This deficiency was compounded by significant elevations in PTH levels, underscoring ongoing disruptions in calcium and vitamin D metabolism. While iron and folate deficiencies showed marked improvement, folate deficiency declined from 46% preoperatively to 12.5%; anemia and vitamin B12 deficiencies exhibited no significant changes. The study also highlighted a troubling decline in adherence to supplementation protocols. Multivitamin use decreased from 92.6% in the first postoperative year to 37% by the fourth year, while vitamin D supplementation dropped from 74.1% to 11.1%. Gender-specific trends were evident, with women more likely to experience anemia and elevated PTH levels, whereas men exhibited higher rates of folate deficiency.

These findings align with broader recommendations from the Obesity Management Task Force (OMTF) of the European Association for the Study of Obesity (EASO). The OMTF advocates for lifelong multivitamins and mineral supplementation tailored to the specific bariatric procedure performed [119]. Regular laboratory evaluations are critical to detect and address nutritional deficiencies promptly, with supplementation adjusted to meet individual needs. In cases of persistent vomiting that disrupt nutritional intake, thiamine should be administered immediately, either orally or via injection, even prior to laboratory confirmation.

The importance of lifelong thiamine supplementation is further underscored by Bahardoust et al., who conducted a meta-analysis of 11 studies involving 1494 patients and found that 27% developed thiamine deficiency postoperatively [120]. These findings highlight the importance of routine follow-ups to monitor dietary intake and ensure adequate nutrient levels, thereby mitigating the risks of long-term complications.

Engebretsen et al. provided additional insights into long-term deficiencies by evaluating the micronutrient status of patients over five years following RYGB [121]. Their findings indicated a substantial increase in iron deficiency, rising from 6% preoperatively to 42% in women and from 0% to 9% in men. Concurrently, anemia prevalence increased significantly, affecting 24% of women and 7% of men by year five.

While the prevalence of vitamin B12 deficiency remained stable, there was notable improvement in folate levels, with deficiency rates dropping from 10% preoperatively to 1% after five years. Despite these improvements, adherence to supplementation protocols varied significantly, 83% continued vitamin B12 supplementation, 65% used multivitamins with folate, but only 25% maintained iron supplementation. Based on these results, the authors emphasized the necessity of long-term use of multivitamins with folate, regular vitamin B12 injections or high-dose oral supplements, and iron supplementation at doses of 45–60 mg/day to mitigate the risk of anemia and other deficiencies [121].

Collectively, these findings underscore the critical importance of lifelong supplementation and structured follow-up protocols to address the metabolic and nutritional challenges inherent in BS. Continuous education on the necessity of supplementation, coupled with standardized regimens tailored to individual patient needs, is essential for optimizing health outcomes and preventing long-term complications. Patients undergoing hypoabsorptive procedures such as RYGB face unique nutritional demands, necessitating enhanced adherence strategies and proactive monitoring. By implementing these measures, clinicians can ensure sustained success in managing the complex nutritional requirements of this patient population.

## 4. Continuous Monitoring of Micronutrient Levels in Bariatric Surgery Patients

Continuous monitoring of micronutrient levels is essential in the postoperative management of BS patients. The anatomical and physiological changes resulting from these procedures increase the risk of nutritional deficiencies, which, if left unaddressed, can lead to severe complications such as anemia, osteoporosis, or neurological disorders [122]. Regular evaluations are critical for identifying both preexisting deficiencies and those that develop postoperatively, facilitating timely interventions. Many patients require long-term supplementation with vitamins and iron, alongside a strict liquid diet during the initial postoperative weeks [123]. Deficiencies, however, can manifest at various postoperative stages, highlighting the importance of individualized care.

While some studies suggest that most micronutrient deficiencies are not highly prevalent during the first-year post-surgery, even with incomplete supplementation adherence [20], these findings emphasize the need for long-term vigilance rather than guaranteeing nutritional stability. This underscores the importance of reexamining supplementation protocols, adopting evidence-based practices, and promoting a multidisciplinary approach. Such an approach should include patient education on the importance of adherence to supplementation protocols tailored to individual needs, alongside regular laboratory evaluations to detect and address deficiencies early. Additionally, ongoing assessments of dietary habits are essential to ensure nutrient adequacy, while proactive identification and treatment of deficiency-related complications are crucial to sustaining the benefits of BS. By integrating these strategies, clinicians can maintain the positive outcomes of BS and significantly enhance the long-term quality of life for patients.

### 4.1. Recommended Frequency of Blood Tests for Different Micronutrients

Regular monitoring of specific nutritional parameters is recommended after BS to detect deficiencies that may not be apparent initially. The American Society for Metabolic and Bariatric Surgery (ASMBS) suggests a structured testing schedule: every three months during the first postoperative year, every six months in the second year, and annually thereafter, regardless of the type of procedure performed [124]. This schedule enables trend analysis over time, facilitating precise adjustments in supplementation and dietary interventions tailored to each patient’s needs. However, not all abnormal blood test results can be attributed solely to the surgery [20].

The ASMBS guidelines suggest testing up to ten key nutrients, with four evaluations during the first postoperative year [9]. The British Obesity and Metabolic Surgery Society recommends a more individualized approach, prioritizing five key nutrients with up to three tests in the first year and reserving others for specific indications or annual follow-ups [122]. Monitoring should prioritize common deficiencies associated with specific bariatric procedures. Routine testing should include key assessments such as liver function, full blood count, ferritin, folate, calcium, vitamin D, and PTH. In cases where symptoms suggest specific deficiencies, such as steatorrhea, night blindness, hair loss, or chronic diarrhea, targeted testing for vitamin A, zinc, and selenium is recommended [46]. A tailored monitoring approach ensures that nutritional deficiencies are identified and addressed promptly. By focusing on procedure-specific risks and incorporating evidence-based testing schedules, clinicians can optimize postoperative care and significantly improve long-term outcomes for bariatric surgery patients.

### 4.2. Role of Healthcare Teams in Patient Follow-Up and Individualized Management Plans

Postoperative nutritional care requires a structured, multidisciplinary approach, with healthcare teams playing a pivotal role in designing and implementing individualized strategies that address the unique needs of each patient [125]. BS is not a standalone intervention but part of an integrated therapeutic process requiring ongoing supervision to maximize weight loss and ensure nutritional adequacy [126]. This comprehensive approach incorporates several critical components, including carefully planned micronutrient supplementation regimens to address procedure-specific risks, promotion of regular physical activity to support weight maintenance and metabolic health, and psychological support to address mental health challenges and encourage adherence to lifestyle changes [127]. Additionally, pharmacological treatments may be necessary to manage comorbid conditions or deficiencies that cannot be resolved through diet alone [128]. A multidisciplinary healthcare team is crucial for monitoring weight loss, assessing mental health, tracking micronutrient levels, and encouraging healthy lifestyle habits that improve the quality of life of these patients [125]. Current guidelines recommend specialized bariatric follow-up for at least two years before transitioning patients to a shared-care model involving primary care providers [125].

### 4.3. Integrating Nutritional Education to Ensure Sustainable Outcomes

BS represents a significant lifestyle change that can be overwhelming for patients due to the preparation, procedure, postoperative recovery, and long-term maintenance requirements. Success depends on a high level of commitment to dietary modifications and the adoption of new habits [125,129]. Nutritional education plays a critical role in this process, enhancing patients’ understanding of dietary recommendations and reinforcing adherence to supplementation regimens. Effective educational programs help reduce the risk of deficiencies, simplify recovery, and facilitate a smoother transition to a healthier lifestyle [129].

Studies have shown that implementing preoperative and postoperative educational programs significantly improves patient’s nutritional knowledge. However, this knowledge can diminish over time without ongoing reinforcement sessions [129]. Nutritionists play a key role in preoperative evaluations, educating patients on micronutrient management protocols, and designing monitoring and supplementation schedules to ensure long-term success [20].

### 4.4. Factors Affecting Patient Adherence to Supplementation Protocols: Behavioral and Socioeconomic Factors

Adherence to supplementation and dietary recommendations is significantly influenced by behavioral and socioeconomic factors, which must be addressed through a multidisciplinary approach [127]. Failure to address these barriers can hinder the prevention of nutritional deficiencies and reduce the overall success of BS [130]. A holistic model, integrating medical and psychosocial factors is better equipped to address patient challenges, such as heightened anxiety when expected results are not achieved. This anxiety can negatively impact emotional well-being and the perception of procedural success [131]. Offering psychological support is essential to identifying and managing emotional or behavioral barriers that could affect individual eating preferences. These are often shaped by early socialization and may require significant shifts in ingrained sociocultural patterns. Thus, continuous psychological support throughout the bariatric intervention process is essential to facilitate habit adoption and provide emotional assistance in coping with post-surgical changes [128,131]. Socioeconomic challenges, such as the high cost of nutritional supplements and limited access to quality healthcare, can further complicate adherence. Research has shown that first-generation immigrants, particularly those from outside Europe, experience less weight loss five years post-surgery compared to other patient groups. This trend persists even after adjusting for common demographic factors such as age, sex, and comorbidities, indicating that suboptimal weight loss is not solely due to biological factors. Challenges such as health literacy, difficulties understanding and applying preoperative information, lack of social support networks, and cultural mismatches between patients and healthcare providers likely contribute to these outcomes [132]. To overcome these barriers, implementing targeted strategies is essential. Tailored preoperative and postoperative educational programs can improve health literacy and empower patients to adhere to supplementation protocols. Providing financial support, such as subsidized access to nutritional supplements and healthcare services, helps alleviate the economic burden faced by many patients. Additionally, building community networks fosters social connections and offers ongoing guidance during the postoperative period. Addressing these challenges ensures equitable access to care, enhances adherence to recommendations, and optimizes the long-term benefits of BS [128,131].

### 4.5. Strategies to Improve Adherence: Patient Education, Counseling, and Support Programs

Educational programs, both individual and group-based, play a central role in improving patient care and adherence to nutritional protocols. Several factors contribute to the efficacy of these sessions, including their comprehensive approach, which enhances participants’ skills in meal preparation and ingredient selection [133]. A study found that patients participating in multiple educational sessions achieved greater BMI reductions and better adherence to nutritional recommendations compared to those with limited participation [134]. Telehealth and mobile health interventions, though not new concepts, have gained popularity in bariatric care. The COVID-19 pandemic accelerated their adoption, helping programs overcome logistical barriers and expand patient access without compromising quality [125,135]. These technologies provide a scalable and cost-effective means of delivering education, counseling, and follow-up care, making them integral to modern bariatric practices.

Continuous monitoring and adherence to supplementation protocols are crucial for addressing the unique nutritional challenges faced by patients undergoing BS. While the anatomical and physiological changes post-surgery necessitate rigorous and individualized care, factors such as behavioral, socioeconomic, and cultural barriers significantly influence long-term adherence.

A robust framework for managing nutritional deficiencies and optimizing outcomes involves several key components. Regular monitoring schedules with structured testing are essential to identify deficiencies and adjust supplementation as needed. Multidisciplinary healthcare teams, including dietitians, psychologists, and medical professionals, collaborate to provide holistic care. Comprehensive preoperative and postoperative educational programs equip patients with practical knowledge and skills, empowering them to adhere to dietary and supplementation protocols.

Persistent challenges, such as suboptimal adherence to supplementation protocols and disparities in access to care, underscore the need for innovative patient support systems. Telehealth solutions can leverage digital platforms to deliver continuous support, education, and follow-up care. Financial assistance programs reduce the economic burden of supplements and healthcare services, improving accessibility. Culturally tailored interventions address linguistic and cultural preferences, enhancing patient engagement and adherence.

By integrating these strategies into patient care, healthcare teams can effectively mitigate barriers and ensure equitable postoperative support. Addressing both clinical and psychosocial aspects of care maximizes the long-term benefits of BS, empowering patients to achieve sustainable health improvements and an enhanced quality of life.

## 5. Conclusions

The prevention and management of MD are essential for ensuring the long-term success of BS. The profound anatomical and physiological changes caused by these procedures predispose patients to significant nutritional risks, including deficiencies in iron, vitamin D, calcium, B vitamins, and fat-soluble vitamins, which can lead to severe complications such as anemia, osteoporosis, and neurological disorders. Proactively addressing these deficiencies through tailored supplementation protocols, regular monitoring, and individualized dietary strategies is critical for enhancing recovery, reducing complications, and improving patients’ overall quality of life.

A multidisciplinary approach is vital in managing these challenges effectively. Healthcare teams, comprising nutritionists, physicians, psychologists, and social workers play a crucial role in providing comprehensive care. This includes implementing preoperative and postoperative nutritional evaluations, designing personalized supplementation regimens, and addressing behavioral and socioeconomic barriers that hinder adherence. Emerging strategies like telehealth, financial assistance programs, and culturally adapted care models offer promising avenues to enhance patient adherence and accessibility, ensuring equitable care for diverse populations.

While the current evidence base on micronutrient supplementation and monitoring provides valuable guidance, it often relies on low-certainty recommendations derived from observational studies or expert consensus. This highlights the pressing need for high-quality, randomized controlled trials to refine dosage recommendations, assess long-term adherence strategies, and evaluate the effectiveness of educational programs. Additionally, research should prioritize understanding variations in nutritional needs across different bariatric procedures and patient demographics to optimize individualized care.

In conclusion, significant progress has been made in the management of micronutrient deficiencies among BS patients. However, persistent gaps in evidence emphasize the need for continued research. By enhancing the evidence base and adopting comprehensive, patient-centered care models, healthcare providers can maximize the benefits of BS, ensuring sustainable health improvements and a better quality of life for patients.

## Figures and Tables

**Table 1:** Preoperative micronutrient supplementation for bariatric surgery candidates.

Micronutrient	Deficiency Implications	Recommended Supplementation
Vitamin D [62,103]	Compromises bone health and immune function; affects over two-thirds of patients.	High-dose cholecalciferol (4000–6000 IU/day) preoperatively, followed by maintenance doses (2000 IU/day). Combined with calcium citrate (1200–1500 mg/day) to prevent secondary hyperparathyroidism.
Calcium [62]	Risk of secondary hyperparathyroidism due to deficiency.	Calcium citrate (1200–1500 mg/day) combined with vitamin D for effective absorption and bone health support.
Iron [46]	Frequently associated with anemia; impacts hemoglobin and oxygen transport.	100–200 mg/day elemental iron with vitamin C (250–500 mg/day) to enhance absorption. Intravenous formulations recommended for patients intolerant to oral iron.
Vitamin B12 [103]	Poor absorption leads to neurological and hematological complications.	Intramuscular injections of 1000 µg every two weeks or high-dose oral supplementation.
Folate [103]	Essential for anemia prevention and hematological stability.	2 mg/day supplementation to address deficiencies, particularly in anemic patients.
Thiamine (Vitamin B1) [46,103]	Prevents severe neurological complications like Wernicke’s encephalopathy.	100 mg/day for patients with poor intake or frequent vomiting.
Fat-Soluble Vitamins (A, E, K) [103]	Deficiencies impact vision, immunity, and coagulation.	Supplementation tailored to individual needs with regular monitoring, especially in hypoabsorptive procedures.

**Table 2:** Immediate postoperative supplementation for bariatric surgery patients.

Nutrient	Recommended Dosage	Special Considerations
Thiamine [46,67]	12 mg/day via multivitamins; increase to 200–300 mg/day in critical cases (e.g., Wernicke’s encephalopathy).	Vital for preventing neurological complications.
Iron [37,46,67]	200 mg/day ferrous sulfate (or equivalent); menstruating women may require double this dose.	Separate iron and calcium supplements for at least two hours to avoid absorption interference. Vitamin C enhances absorption.
Zinc [46,67]	15 mg/day for all patients; increase to 30 mg/day for BPD/DS patients.	Maintain appropriate zinc-to-copper ratio (15 mg zinc to 2 mg copper).
Copper [46]	2 mg/day	Included in multivitamin formulations; important for enzymatic functions.
Vitamin D [46,63,118]	2000–4000 IU/day	Adjust doses upward for BPD/DS patients to maintain serum levels > 75 nmol/L.
Calcium [46,67]	1200–1500 mg/day for SG and RYGB; 2400 mg/day for BPD/DS.	Use calcium citrate for better absorption; avoid concurrent iron supplementation.
Vitamin B12 [46,67]	Intramuscular injections of 1 mg every 3 months; increase frequency if neurological symptoms develop.	Critical for neurological and hematological health.
Vitamin A [45,46]	10,000 IU/day; increase to 50,000 IU/day for BPD/DS patients if necessary.	Adjust doses based on clinical monitoring.
Vitamin E [115]	60 IU/day	Important antioxidant; more monitoring needed for BPD/DS patients.
Vitamin K [115]	300 µg/day for BPD/DS patients.	Use water-miscible formulations for better absorption in hypoabsorptive surgeries.
Selenium [45,117]	Adjust based on deficiency detection through regular monitoring.	Universally recommended for all bariatric surgery patients.
